# Supplementary material for: MetaRibo-Seq measures translation in microbiomes
Source: Nat Commun. 2020 Jun 29;11:3268. doi: 10.1038/s41467-020-17081-z (PMC7324362; doi:10.1038/s41467-020-17081-z)
Supplement: Supplementary file 10 — Supplementary Data 7 [file 41467_2020_17081_MOESM10_ESM.zip › File2/Confidence_High_Taxonomy/157270_out.krona.html]

Javascript must be enabled to view this page.

members
magnitude
magnitudeUnassigned
count
unassigned
taxon
rank

157270\_out

7

SRS098571\_contig\_number\_contig-100\_279.368809
1

superkingdom
2157
2

28890
phylum
2

2
183963
class

2
1644060
order

2
1644061
family

genus
121871
2


SRS053356\_contig\_number\_23231SRS1041145\_contig\_number\_29238
2
588898
species

2
superkingdom
2

phylum
1224
2

subphylum
68525
2

28221
class
2

29
order
2

2026763
species
2

SRS023914\_contig\_number\_contig-100\_2533.123578SRS051031\_contig\_number\_contig-100\_4143.162572


SRS013687\_contig\_number\_contig-100\_5292.145266SRS076929\_contig\_number\_contig-100\_3149.124224
2
